# Exploring English speaking Muslim women’s first-time maternity experiences: a qualitative longitudinal interview study

**DOI:** 10.1186/s12884-019-2302-y

**Published:** 2019-05-06

**Authors:** Shaima Mohamed Hassan, Conan Leavey, Jane S. Rooney

**Affiliations:** 1 Department of Health Services Research, NIHR CLAHRC North West Coast. Institute of Psychology, Health and Society, University of Liverpool, Waterhouse Building, Brownlow street, Liverpool, L69 3GL England; 2 Faculty of Education, Health and Community. Public Health Institute, Liverpool John Moores University, Liverpool, England; 3 Faculty of Education, Health and Community. School of Nursing and Allied Health, Liverpool John Moores University, Liverpool, England

**Keywords:** Cultural competency, Maternity, Midwifery, Motherhood, Muslim women, Islam

## Abstract

**Background:**

Muslim women of child-bearing age make up a fair part of the UK society, however, literature addressing their health needs or experiences of health services have not been extensively researched. The term ‘Muslim’ is often combined with ethnic group identity, rather than used to refer to people distinguished by beliefs or affiliations. Muslim women commonly observe certain religious and cultural practices during their maternity journey. The little research there is in this area suggests that more could be done from a service provision perspective to support Muslim women through this significant life event. The aim of this study was to investigate Muslim women’s perceived needs and the factors that influence their health seeking decisions when engaging with maternity services located in North-West of England.

**Methods:**

The study used longitudinal semi-structured interviews with seven English-speaking first-time pregnant Muslim women receiving maternity care in North-West of England. Total of 21 interview; each woman was interviewed during the antenatal (29 to 40 weeks of pregnancy), immediate postnatal (within the first 2 months after birth) and later postnatal (4 months after birth) period. Audio-recorded interviews were transcribed and thematically analysed using Braun & Clark (2006) as a guide to forming a systematic approach to handling raw data.

**Results:**

Muslim women associated most aspects of the maternity journey with their religious beliefs. Religion was not the primary reason for them becoming pregnant, yet it was an aspiration for them becoming mothers. Emerging themes include: 1) a spiritual perspective; 2) expression of religious requirements; 3) perceptions of healthcare professionals. Religious values and practices provided a positive resource for women during their maternity journey. They described how healthcare professionals approached their needs, while highlighting their concerns of the negative presentation of Muslims in Western media.

**Conclusion:**

Muslim women need to feel confident to express their needs within a maternity setting. Lack of awareness amongst healthcare professionals around religious values and how Muslim women may feel when expressing their needs can inhibit them getting optimal care that acknowledges their needs. The study concludes that educating healthcare professionals about Muslim women’s worldview would enhance the quality of maternity care for Muslim women.

## Background

Childbirth is the single largest cause of women’s admission to NHS hospitals in England [1]. Whilst most women report positive experiences of maternity care, existing evidence suggests that women from Black and Minority Ethnic (BME) groups in the UK experience poorer pregnancy outcomes, poorer maternity care, higher risk of adverse perinatal outcomes and higher maternal morbidity than the resident White women [2–7]. Creating striking inequalities persist in neonatal and infant outcomes between white and ethnic minority groups in the UK with some groups being particularly disadvantaged [2].

Ethnic groups in the UK are generally differentiated by a combination of factors including racial origin, skin colour, cultural and religious affiliation, national and regional origins and language [2]. Religion has been recognised as a key element of the UK’s BME population identity in contrast to the UK white population [8], with the Muslim population making up the second largest religious group in the UK [9].

People from different ethnic groups have different cultures, religions and beliefs that influence the way they see, behave and react to the world [10]. These factors are powerful filters through which the individual receives information (such as belief systems, religion and cultural values) [11]. Understanding such attributes can help in the development of culturally sensitive maternity services.

This study focused on exploring Muslim women as a unique group. For most Muslims, Islamic beliefs and practice dominate aspects of their individual life and behaviour; it represents the prism through which Muslims view and interpret their world [12, 13]. Islamic beliefs not only provide guidance in spiritual matters but also place considerable emphasis on health. There are a number of Islamic beliefs that will influence the attitudes and behaviours of Muslim patients in hospitals and community settings, such as beliefs about modesty, privacy, dietary restrictions, and fasting [12]. Due to their particular religious and cultural beliefs, Muslim women face barriers in accessing and utilising healthcare. Many providers also feel challenged in meeting the needs of Muslim patients, especially female Muslim patients [14].

Only a handful of studies focus specifically on Muslim women’s experiences of culturally appropriate and patient-centred care, including maternity services [14–19]. The available evidence suggests that many Muslim women receive poor and inappropriate maternity care, which puts them and their babies at risk [3, 5]. Muslim women encounter poor communication, stereotyping, racism, inaccurate cultural assumptions held by some practitioners, and a general lack of research and sensitivity concerning the cultural and linguistic needs of women from diverse populations [16, 17, 20]. Studies conclude that services could do more to support Muslim women through this spiritually and culturally significant life event [15, 20].

This suggests a general lack of understanding of the particular needs of religious and ethnic communities among academics, policymakers and clinicians, as without an understanding of these needs they are in no position to address them [13]. Improving the healthcare experiences of populations from disadvantaged minority ethnic groups requires policymakers and healthcare practitioners to understand when cultural context makes a difference and when it does not [20]. This depends on the education, professional confidence, and competence of midwives, obstetricians and general practitioners in understanding and acknowledging the needs of religious and ethnic communities [21]. Therefore, much work is needed to create research evidence on current specific maternity interventions for BME women in the UK. This will enable policy makers modify services and develop services, which can reduce inequalities and improve maternal and birth outcomes [22].

This study therefore aimed to generate insight into Muslim women’s maternity experiences, consider their access to and experiences of the NHS maternity services, and the religious factors that influence their health seeking decisions. Greater understanding of this phenomenon will provide an opportunity for maternity services to deliver the best possible care for this client group and endeavour to meet their cultural and spiritual needs.

## Method

This paper presents findings from the first phase of a three-phase qualitative PhD study exploring English speaking Muslim women’s experiences of motherhood while engaging with NHS maternity services [23]. Qualitative research adept at exploring phenomena that are under investigated or have not been explored before, through generating in-depth data and a thorough understanding [24]. Its application in this study enabled the research to create an in-depth insight of the maternity experience as told from Muslim women’s own perspective.

### Research design

This study used qualitative longitudinal in-depth semi-structured interviews with seven Muslim women experiencing maternity for the first time in the UK. The strength of this approach is that it is useful in capturing a phenomenon that involves a developmental process [25]. Rather than providing a snapshot of the women’s maternity journey, longitudinal interviews provided an insight to how motherhood unfolded over time for each participant.

In total, 21 interviews were conducted, each Muslim woman was interviewed during the antenatal period (29 to 40 weeks of pregnancy); during the immediate postnatal period (within the first 2 months after birth); and finally during the late postnatal (4 months after birth) period. Interviewing Muslim women at these significant periods allowed them to express their experiences over time, as and when they encountered different healthcare professionals and services in relation to different aspects of their maternity journey.

(SH) conducted interviews in women’s homes to enhance them feeling confident and in control of the environment that they were in. The duration of each interview ranged from 60 to 75 min. Based on the review of the literature, an interview guide was developed. The interview guide was divided into three sections to complement the three stages of the longitudinal interviews [23]. First interview asked general questions around the experiences of pregnancy, access to services, services’ ability in meeting needs, religious practices, needs during pregnancy, and religious needs that are specific to birth. The second interview asked questions around the experiences of labour, service access, after labour care, if they had been able to implement their specific religious practice at birth and how they had been addressed. Finally, the last interview asked questions about the overall experience of pregnancy and birth, service provision and implications of service provision [23].

Eligible Muslim women were provided with the study’s information sheet, highlighting the aim of the study and what it involves. Those who were willing to participate signed a consent form to confirm their approval. While providing consent, participants were provided with a simple demographic questionnaire with a set of questions including age, ethnicity, marital status, level of education, employment statues and place of birth. Interviews were conducted in English and all interviews were audio recorded.

### Participant and recruitment

The study used purposeful sampling to recruit first time pregnant Muslim women living and receiving maternity care in the Merseyside region of England. To identify study’s participants, (SH) a female researcher, directly approached Muslim women who were recognisably pregnant in a local mosque in Merseyside. The researcher was familiar with the mosque’s setting as a place of worship but also as community centre and a social hub used by many local Muslims. The researcher recognised that approaching participants at the local mosque might limit the study to participants that attend such place. Therefore, the researcher also approach women directly through existing local Muslim women community groups, such as the Mother & Toddler group and Somali women breastfeeding group. Through conversation, the researcher was able to inquire if it was the woman’s first time maternity experience. First-time pregnant Muslim women who reported themselves as followers of the Islamic religion were invited to participate if they were age 18 years and above; were receiving antenatal care, and spoke fluent English. Having a language barrier is a significant factor that has a great impact on an individual’s experiences and can be the barrier to effective and equitable healthcare [26]. Women’s experiences with language barriers may have a different dimension to the experiences of women with no language barriers. This study’s insight into the maternity experiences of English speaking Muslim women in the UK will support future exploring of the maternity experiences of non-English speaking Muslim women.

Even though this study selected Muslim women living in one geographical range for convenient access, the researcher strove to select Muslim women who will reflect variation (ethnicity, age, education, marital statues) in the sample of this study. The sample included Muslim women between the age 20 to 33, UK born (4) and born outside the UK (3), housewives (4), employed (3), married (7), ethnicity; Yemeni (3), White British (2), Somali and British Indian, and with secondary school as minimum level of education (7).

Data were collected until no new, or repetitive, information emerged from the interviews. The early triangulation process of data analysis and the identification of initial patterns indicated that there was no new emerging information. Although data saturation was achieved with six women, one more woman was interviewed to ensure that no new information was emerging.

### Data analysis

Audio recorded interviews were transcribed by the researcher (SH); (CL) and (JR) reviewed the transcripts and audio recordings to ensure consistency and credibility of the analysis process. A thematic analysis approach was used to aid the researcher in identifying, analysing and reporting patterns within data sets [27]. This approach started with the researcher reading all the transcripts repeatedly to enhance familiarisation and to develop an in-depth understanding of the data.

The (SH) employed a manual method (using Excel and Words software) to manage and analyse all the data. This manual process requires the researcher to manage and analysis the data early to avoid missing critical evidence and provide trustworthiness in the process [28]. The researcher did this as the study’s triangulation process of data collection required it. Following each interview, the researcher made notes of certain points that needed to be explored further in following interviews, highlighting what was meaningful and relevant to the research. Revisiting data and exploring the initial ideas helped in identifying clear possible patterns that are relevant to the research question.

The data was organised into potential themes by grouping similar sets of data connected to each theme [27, 29]. The researcher then started to think about the relationships between the themes, to identify the main overarching themes and sub-themes within them. The researcher illustrates the themes identified below with rich, verbatim extracts expressed by a number of participants.

### Ethical consideration

Ethical approval for the study was obtained from the NHS Research Ethics Committee (through the Integrated Research Application System (IRAS) prior to commencing data collection.

Written and informed ‘process consent’ was considered throughout this study. Participants were interviewed at three stages; to ensure that participants were still interested in being part of this study, following each interview, participants were asked if they were still happy to later be contacted regarding the next interview. In addition, at the start of subsequent interviews participants were reminded of the overall aim of the study and the researcher obtained verbal and written consent, emphasising their right to withdraw from the study at any point.

To ensure researcher’s safety when travelling and conducting interviews at participants homes. Researcher ensured that a colleague is aware of their whereabouts; expected time of completing an interview and an action plan in place if researcher has not contacted after expected time of interview session completion.

Any identifying information was removed from transcripts prior to analysis to ensure anonymity.

## Findings

The characteristic of the women who were interviewed are described above, however for the purpose of the study the women are identified as Noor, Hanan, Khadija, Sahar, Eman, Nesreen and Fatimah (Pseudonyms).

### A spiritual perspective

Mothers perceived their motherhood experience through the lens of Islamic teaching. Words such as ‘*gift*’, ‘*blessing*’, ‘*reward*’, ‘*worship*’, ‘*heaven*’, ‘*lofty*’, ‘*Alhamdolilah*’ (All prise to *Allah*), ‘*fate*’, ‘*acceptances*’, ‘*Inshallah*’ (in *Allah’s* will), ‘*obligations*’ and ‘*Sunnah*’ (Prophetic traditions) echoed across women’s narratives. This Islamic perspective enriched women’s experience of pregnancy and birth. The condition of pregnancy was regarded as a spiritual state, irrespective of whether a woman actively practises her religion:

*“Being a Muslim, there is a real focus on motherhood that is not just attached to being a mother, but the actual pregnancy... I think it is a really humbling experience because you realise that your rank is just so high, just because you carry this child… You have a difficult day and you say to yourself, ‘you know what, I am getting rewarded for it’. I think that is reassurance all the way… I think that religion gives you that boost and I don’t think you can get that elsewhere.”* (**Noor**)

The value that Islamic teaching surrounding motherhood was one of the reasons for Muslim women aspired to becoming mothers. However, their belief in pregnancy being *Allah’s* (God) giving ‘gift’ and it is received through *Allah’s* will, made Muslim women who are trying to become pregnant feel content and did not feel the need to haste into seeking medical intervention.

“*I was not using anything, but it didn’t happen, it is from Allah when it is meant to happen… it is a gift from Allah, without Allah nothing is possible. You know that it is in Allah’s hand when you are meant to have that baby and it is in Allah’s hand if you are meant to be a mother. So I believe in that, it was meant to happen to me now*.” (**Khadija**)

This tenet of the Islamic faith helped women to stay optimistic and resilient when faced with struggles during pregnancy, labour and postnatally. Muslim women reported how they tried to face challenges with patience and called for spiritual intervention through prayer, *Dua’a* (supplications), or by calling on *Allah’s* name and reciting the Quran.

“*I think without my faith I would probably lose a lot of confidence. I would probably go through depression or something because of the things I went through. I experienced a lot of things in my pregnancy… (Postnatal) I felt like I was being unthankful ‘why am I sad when I have this blessing in my hand?’ So I read a lot of Quran and I played a recording of it while sleeping with my baby and just got through it that way, I think it helped me calm down and I think baby blues are physical and I used spirituality to help me out*” *(****Hanan****)*

“*I was in a lot of pain and I was in and out of the hospital when I was 16 weeks… I try to think about how Heaven is under the feet of a mother and my husband will talk to me about the [God’s} rewards of the pain and it does help to lift me up and it gets me out of it straight away*.” (**Nesreen**)

Meanwhile, Muslim women did not rely only on their faith when faced with challenges, they also regularly attended antenatal appointments, contacted their midwife, GP and emergency services when needed. They reported that seeking medical interventions also has a religious dimension, as it is a religious duty to look after one’s self and this does not contradict reliance on *Allah* or the acceptances of one’s fate.

*“I went to A&E, and they checked everything and said that everything was fine with the baby and ‘the bleeding was just the walls of your inside’ I was reassured and I had a scan and then I had my midwife appointment. My doctor referred me to the antenatal care where I had my first midwife appointment in the GP. I just went there to make sure that everything was okay with the bleeding, it was minor bleeding but I wanted to be reassured, just after I had that, I felt better” (****Hanan***)

### Expression of religious requirements

The second theme focused on women’s difficulties expressing their religious requirements when engaging with maternity services. Even though the spiritual dimension was a resource for Muslim women, medical practices and Islamic practices could sometimes conflict. For example, women felt strongly toward Down Syndrome (DS) screening, since their belief in a child being a gift from *Allah* meant that terminating the pregnancy was only an option when a mother’s life is at risk. Some women avoided discussing the DS screening and any information provided by midwives, so as to avoid creating anxiety. When offered DS screening, women quickly declined without expressing their concerns or giving the midwife the chances to explain the process of the screening to support informed decision-making. Only Khadija had the discussion with her midwife, which helped her understand that DS screening is also about informing the mother and dose not necessary need to end with the termination of the pregnancy.

*“I thought I do not want to put fear in me of what could happen. If the baby has Downs [DS] it is what God has given me. Abortion could not even enter my mind…what will be, will be.”* (**Sahar**)

“*You have gratitude to Allah as it is in his hands, regardless of whether your baby is well or not, but in the other hand it is good to know and prepare for it. When I was asked if I want the screening, it made me think ‘do I really need to know?’ because it is like obvious you do not want anything to happen to your child and you can refuse to have the screening. The midwife explained to me and said "I would rather you have it than not to have it, then you will know if there is anything wrong with you or the baby*”. (**Khadija**)

Muslim women also worried about preserving their modesty during labour and/or examinations, particularly in the presence of a male healthcare professional. Women felt anxious over raising these issues, especially requesting all female staff. They anticipated for their requests to either be dismissed or create a burden on over-worked healthcare professionals.

*“The nurse said that ‘a doctor will come to see you’. Automatically, I thought, it is going to be a man. Sometimes you do not want to be fussy, like, they would think, ‘the Muslim girls are the fussy ones because they do not want a male doctor’ and that the staff would have to run around finding a female for us. So I felt ashamed because it was a serious situation and they just wanted the doctor to see me. So I was just praying in my head, ‘Please let the doctor be a woman’ and, Alhamdulillah, it was a female doctor.” (* ***Eman*** *)*

Furthermore, women explained that along with great religious ‘*reward*’, motherhood carries great religious responsibilities. Encouraging parents to ensure not only the welfare of their child but also their own welfare. As part of this, pharmaceuticals of animal origin present Muslim women with a serious dilemma and cause them to weigh their health and their child’s health against their religious principles. Certain materials are forbidden to Muslims, such as any products that derive from cattle not slaughtered according to Islamic practice, or any material from pigs. They felt it was important to be aware when such proscribed derivatives were in medicines and supplements, such as infant Vitamin-K injections, to enhance informed choice. Yet, Muslim women did not express the importance of this religious need to their midwives. Rather, some made the assumption that healthcare professionals are aware of their dietary requirements and would inform them if certain medications did not comply with their religious beliefs. Other women took it on themselves to explore and sought this information from other avenues and not their midwives.

*“I found out that the Vitamin-K actually has pig’s ingredients, which is completely prohibited in [my] religion. For me it was an informed decision not to give it to my child because there are actually other options available that are vegetarian based. A lot of doctors and nurses do not tell you about the injection, I think little details like that need to be told, especially to Muslims, because pig is such a big thing for them and to have it injected into their child who has just came into the world is just so wrong! I think if I had not come across this information of the injection and I found out later after giving it to my child, it would have been quite heart breaking.” (* ***Noor*** *)*

*“If there was something that is forbidden in other people’s religious or dietary needs, I think they are smart enough to tell us and it would be silly if they do not. I know that the products are given for the benefit of the child but if there was something I knew of that is unlawful then I would not give it to my child*”. (***Hanan***)

Another practice that some Muslim women were anxious and avoided discussion with healthcare professionals, was male circumcision. It is an obligation for a baby boy to be circumcised in the Islamic tradition. This practice presented challenging for Muslim women, they expressed great trust in NHS services, yet feared to discuss such practice with their GPs or midwives. Instead some searched for NHS credited private clinics for their child’s circumcision. Muslim women did not have the confident to discuss such practice, they believed that healthcare professionals will considered it as taboo.

“*There are a few NHS approved clinics that do it…So we searched online and we didn’t speak to any medical staff or midwives because I know there is a lot of controversy about it and it’s something that you sort out yourself…I think it is a difficult one because it’s like a taboo subject to mention and they think it is just cruel”.* (***Noor***)

### Perceptions of healthcare professionals

The third theme focuses on women’s assumptions that healthcare professionals having a negative views of Islamic birthing practices, Muslim women and Islam in general. For most women this was not caused by negative encounter during their care, but by a more general concern over Islam and Muslims portrayal in Western media. This diminished women’s confidence in discussing religious values and practices with healthcare professionals because they assumed that they would be viewed at odds with Western norms. One women, Sahar, was complimented by her midwife for looking “*nice… like a modernized Muslim*”:

*“She [the midwife] said ‘We had one woman wearing a veil; I do not like them, the full veil scares me. She painted this picture of this vulnerable little woman cowering, face covered…It just annoyed me because prior to that she came across as down to earth.*” (***Sahar***)

Muslim women felt that they had to explain themselves every time their religious beliefs and practices were mentioned. Rather than just asking for what they need, they felt the need to explain why for example they wanted to be seen by a female healthcare professionals. While others found it easier to ‘hide’ their religious practices rather than discussing them freely. Even though they wanted a healthcare professional’s opinion on certain religious practices during pregnancy, such as fasting during pregnancy. Most women avoided discussing this with their midwives, even so, some women who mentioned Ramadhan, found themselves being ‘*told off’* by midwives for fasting without indicating that they were fasting or intended to fast.

*“Honestly people just hear the word fasting and they [health professionals] think that you are so extreme …I remember the first time I had a check-up during Ramadhan, my blood pressure was low and I was fasting that day. I did not tell her that I was fasting because I just knew it was going to bring this thing about religion, like, ‘Your religion makes you fast even when you are pregnant?’” (* ***Noor*** *)*

*“I would have felt gutted and really upset if I had told the midwife that I intend to fast and she was to discourages it because fasting for Muslims is just so important and to even attempt to make it as a negative thing you could not have caused someone more events really. Some people make assumptions and judgments about our religious practice and you constantly have to defend yourself”. (* ***Sahar*** *)*

For some Muslim women who vocalise their religious needs, felt that because of the lack of understanding of religious values amongst healthcare professionals, their need were not appropriately acknowledged and at times were dismissive of their choices.

“*I breast fed at first, it was difficult because when you are on the ward and it was visiting time, I you had to draw the curtains and if someone comes in I had to cover myself with a scarf...In the morning when they come in they open the curtains and the window, the non-Muslim women are okay to breastfeed but me I have to draw the curtains and then breastfeed. That was a struggle and then I told the midwife ‘please don’t open the curtains because I am breastfeeding and I don’t want anyone to see me’ but she will say ‘okay’ and then they change shifts so the other midwife comes and then I had to explain again, you just keep telling them.”* (***Khadija***)

*“Once there was a male student and before the midwife called me in to the room she came out and asked me if I would be okay with a male student in the room. I said to her ‘no’ and she still asked me if it is okay if he can check your tummy and I said ‘no’. When I walked into the room the male student was there and she asked me again if it was okay for him to be in the room and I was like ‘uh my God, I already told you outside’ I felt embarrassed. She was like ‘okay’ and she most of thought I would back out of it and change my mind but I still said ‘no’. Even though I told her I do not want him to be in the room and I try to explain it to her, she just makes it out that she is in another world. [Male student remained in the room] So I just let her be and when she wanted to check my tummy, I told her to close the curtains, then she listened”. (* ***Eman*** *)*

Despite every woman being given the opportunity to discuss and write a birth plan (a record of what the pregnant woman would like to happen during labour and after the birth), which includes her choice of pain relief, place of birth and any specific practice that she would like the midwife to be aware of. The majority of Muslim women were not confident in discussing religious practices that they intended to implement during their labour. Most were not aware that discussing religious practice is an option within the birth plan and felt that there named midwife during labour would not have the capacity to read the birth plan. Most women relayed on family members to assure religious practice during labour, yet doing this was also challenging for some.

“*I do not think they even read the birth plan that you write... Even the birth plan appointment was very short and it was like a check list asking me about the types of pain relief that I would want and simple things. But I did not mention that I wanted a silence birth because midwife did not ask and I do not know that I can even discuss it with her…I thought it was a religious thing and my family will do that, so no need to mention it*”. (***Fatima***)

“*I said something like ‘we need to read something for the baby can you please be quiet while we do that’ they just said ‘okay’ and they just continued talking and whispering. It was clear that they did not understand what we were doing. When they both left the room he [husband] was able to read it into both of her [child] ears. If they knew they would have said ‘they want to do their practice now, so be quiet’ but they were not aware.”(****Sahar***)

## Discussion

This study focused on the maternity experiences of Muslim women as a heterogonous group with diverse backgrounds and explore the factors that influenced their health needs and health seeking-decisions when engaging with maternity services in the North West of England.

The study shows that Islamic beliefs and practice with roots in the Quran (foundation of Islamic law) and *Sunnah* (Prophetic traditions) were at the core of Muslim women’s maternity experiences. They often used the terms ‘*Muslim culture’*, ‘*Islamic culture’* and ‘*my/our culture’* when talking about their religious beliefs and practices. In accordance with previous studies, the study emphasizes that religion is embedded in the inner life and social behaviour of individuals and gives individuals meaning in their lives and validates their lifestyle [12, 13].

For Muslim women, becoming a mother is an act of worship that accords mothers a lofty position in the sight of *Allah* (God) and great respect within the community. Even if the Muslim woman does no more than simply bring her child into this world. Muslim women believed that this spiritual experience was independent of their level of religiosity, a divine meaning is woven into the act of motherhood itself. These spiritual dimension was a resource that played a significant role in the women’s experiences, helping them stay positive, optimistic and resilient when faced with struggles during pregnancy, labour and postnatally. When faced with significant challenges Muslim patients (practising and non-practising) generally called for spiritual intervention [30]. Likewise Muslim women in this study, experienced physical comfort after making *Dua’a* (supplication) to *Allah* and regained confidence both physically and mentally.

Meanwhile, when engaging with maternity healthcare services, Muslim women sometimes lacked confidence discussing their religious needs, such as fasting, the need for a female healthcare professional and concerns over clinical practices, such as DS screening and Vitamin K injections. To exclude the religious aspect or separate it from spiritual needs could be detrimental to some individuals [31] and this is most certainly the case for Muslim women in this study, simply because their spiritual needs are religious in nature. The study suggests that separating Muslim women’s spirituality from religion can be almost impossible, it is suggested that both spiritual and religious aspects of care should be supported rather than trying to separate the two [32]. Rather than specifying a spiritual or religious approach to care, this study supports the notion of a holistic approach to care that recognises that spirituality and health are very much intertwined for most individuals.

Recognising this will help give Muslim women control to make choices; healthcare providers do not necessarily need to be religious but need to have a broad view of spirituality that accommodates diverse views [33]. This is also important when recognising Muslim women’s perceptions of healthcare professionals. Muslim women in this study expressed their awareness and concerns of the impact of Western media and it portrayal of Islam and Muslims in general. A study assessed the portrayal of Muslims in the British print media between 2001 and 2012 and concluded that Muslims are typically cast in a predominantly negative light and are depicted in a substantially more negative way when compared to analogous groups [34]. Against this backdrop, Muslim women in the Western world tend to be portrayed as victims and oppressed and the face veil (*burqa*) has long been used as symbol of oppression and the patriarchy of the Islamic world [35, 36]. This image has run through the media, politics, arts and literature, even though it is estimated that 90% of Muslim women world do not wear the *burqa* even in most Muslim countries [36]. In addition, debates and policy in Europe about banning or regulating wearing the veil contribute to the assumptions that if Muslim women wearing Islamic garments had a choice they would not wear headscarves, face veil (*burqa*) or any such clothing. Therefore, the prevailing discourse is that Muslim women are oppressed or even enslaved and need to be saved or forcibly emancipated [36].

Due to this Muslim women felt they needed to negate and/or not add to such negative images of Islam and made an effort to present and explain their religious practices to help avoid misconceptions or misunderstandings. However, Muslim women in this study reported that they would not discuss and may hid certain religious practices from healthcare professionals if they thought that healthcare professionals might view them as different or at odds with Western norms. This is a serious issue that makes building better relationships between Muslim women and healthcare professionals difficult. Therefore, one cannot overlook directly or indirectly the role of the Western media in portraying negative images of Islam and in particular Muslim women [34, 35], and its effect on Muslim women’s maternity experience in the current study. Hence, it is important when developing a competence model of care that is appropriate for Muslim women and other religious groups to recognise that culture is inseparable from the political-economic climate that we live in [37].

It is important to provide care that is all-inclusive and accepting of differences and that is competent in creating an atmosphere where all women with different backgrounds can discuss spirituality. This study agrees that cultural competency is not about learning the language or adopting the cultural values of a patient, but about respecting differences and making sure that these are bridgeable in order that they do not negatively affect the process of care [38].

### Strengths and limitations

This is the first qualitative longitudinal interview study that explored English speaking Muslim women’s first-time maternity experiences in the North West of England. The findings provides a unique insight into the experiences of Muslim women when engaging with NHS maternity services and how religious values were key assets to their experiences’. Women who participated in this study reflected the variation of English speaking Muslim women experiencing maternity care in the North West of England. The aim of capturing these experiences through the narratives of seven Muslim women was not to generalise but rather to create in-depth contextualised understanding of these experiences. This study expands the knowledge and understanding of Muslim women’s maternity care, which will help in further development of maternal services competency in addressing the needs of Muslim women.

The primary limitations of this study are the approaches adopted for recruitment. The participants were recruited from only one geographical diverse area in the North West of England. The purposeful sampling from the local mosque and local Muslim community groups may have further limited the outreach to other potential participants. Possible other avenues that could be used for recruitment would be local children centres, ethnic community centres, local multi ethnic community groups, and through the Trust or General Practitioner surgeries.

## Conclusion

The connection between religious values, religious identity and maternal care has not previously been discussed for Muslim women. This study makes an important contribution to the wider understanding of Muslim women’s opinions of motherhood and their experience of NHS maternity services. It recognises that spirituality is intertwined with Muslim women’s motherhood experiences and this is an important lesson for policy makers and healthcare professionals. Muslim women’s religion is a comfort and resource, yet it can also be a source of contention and anxiety in a maternity setting especially when women feel misunderstood and stigmatised for their beliefs. It is important that maternity services support the development of policies and work force to recognise the importance of women’s value systems and to enhance acceptance of differences to create an atmosphere where women can feel confident to discuss their specific needs.

## Abbreviations

BME: Black and Minority Ethnics
DS screening: Down syndrome screening
IRAS: Integrated Research Application System
